# Vision-related quality of life compared to generic measures in retinoblastoma survivors

**DOI:** 10.1007/s11136-022-03315-8

**Published:** 2022-12-15

**Authors:** Paula J. Belson, Nancy A. Pike, Jo-Ann Eastwood, Mary-Lynn Brecht, Jesse L. Berry, Ron D. Hays

**Affiliations:** 1 Department of Anesthesiology and Critical Care Medicine, Children’s Hospital Los Angeles, 4650 Sunset Blvd. Mailstop #12, CA 90027 Los Angeles, USA; 2 School of Nursing, University of California, Los Angeles, CA USA; 3 USC Roski Eye Institute, University of Southern California Keck School of Medicine, Los Angeles, CA USA; 4 RAND Corporation, Santa Monica, CA USA; 5 School of Medicine, School of Public Health, University of California. Los Angeles, Los Angeles, USA

**Keywords:** Health-Related Quality of Life, Vision-Related Quality of Life, Retinoblastoma, PROMIS®-29 Profile, NIH Toolbox® VRQOL

## Abstract

**Purpose:**

To (1) Compare vision-related quality of life (VRQOL) in adolescent and young adult (AYA) unilateral versus bilateral retinoblastoma (RB) survivors using a vision-targeted measure and a generic health-related quality of life (HRQOL) measure and (2) Assess associations among VRQOL and generic HRQOL domains and overall QOL and estimate associations of the VRQOL and HRQOL domains with overall QOL.

**Methods:**

The National Institute for Health (NIH) Toolbox® VRQOL instrument, PROMIS®-29 Profile v 2.1, and a single-item QOL measure were administered in a cross-sectional study of 101 RB survivors. Reliability for multi-item scales was estimated. Product-moment and Spearman rank correlation coefficients and stepwise ordinary least squares were used to measure associations of other variables with overall QOL.

**Results:**

Significantly worse VRQOL was reported by bilateral than unilateral RB survivors. Cronbach’s alpha coefficients for all VRQOL scales ranged from 0.83 to 0.95. Medium to large correlations were found between all NIH Toolbox® VRQOL scales and the PROMIS®-29 measures. Depression and ability to participate in social roles and activities from the PROMIS®-29 Profile accounted for 38% of the variance in overall QOL with the psychosocial domain of the NIH Toolbox® VRQOL explaining 16% of the variance.

**Conclusion:**

VRQOL is impaired in bilateral RB survivors. VRQOL is associated substantially with the PROMIS-29 generic HRQOL measure but has significant unique associations with overall QOL. The NIH Toolbox® VRQOL measure provides important information about the vision-related effects on daily life of AYA RB survivors.

## Introduction

Retinoblastoma (RB) is an intraocular (eye) cancer that forms in the developing retina in very young children and can affect one or both eyes [1]. The yearly worldwide incidence is one in every 16,000 live births [1] with an improved survival rate of 97% over the past decade in the United States (U.S.) [2]. Treatment depends on the size, location, and laterality of the tumor(s), which can be unilateral or bilateral, resulting from spontaneous or hereditary gene mutation [3]. The number one treatment goal in the management of RB is to save the life of the child; secondary treatment goals include vision preservation and ocular salvage. Nonetheless, survivors can be left monocular (unilateral), visually impaired, or blind. RB affects physical, mental, and social health aspects of life. Hence, studies examining health-related quality of life (HRQOL) in RB survivors are needed but to date have been limited [4–6] with only one previous study using a vision-related quality of life (VRQOL) measure [7].

Assessing HRQOL is important during RB treatment due to decisions negotiated between the provider and the parent. Many parents are hesitant to accept enucleation (surgical removal of the eye), a common treatment for RB, due to fear of social stigma and cultural factors, instead opting for alternate, possibly less-effective therapies [8]. This can result in tumor metastases and not infrequently eventual enucleation after failed attempts at globe salvage. These decisions are even more difficult when both eyes are affected. Knowledge of HRQOL in RB survivors can be utilized by providers in discussions with parents during early treatment decision making.

Results of HRQOL studies of RB survivors have found varied results that may reflect the use of a variety of measures [4]. Generic HRQOL measures are useful to compare disease groups with healthy counterparts. However, sensitivity to specific HRQOL effects of the disease may be lacking. There is no disease-targeted HRQOL measure that has been psychometrically evaluated for use in the RB population [5]. Limitations in the methodologic quality of pediatric ophthalmology and oncology measures and applicability of content to the RB population have been identified [5]. However, it is uncertain how well a generic HRQOL measure captures the impact of RB compared to a VRQOL measure.

In this study, we compare VRQOL in adolescent and young adult (AYA) unilateral versus bilateral RB survivors. We hypothesize that VRQOL will be worse for those with bilateral than unilateral RB due to more extensive treatment and visual impairment with bilateral disease. In addition, we assess associations among the VRQOL, generic HRQOL, and overall QOL.

## Methods

### Design, setting, and sample

As this is a secondary analysis of an existing data set [9], no power analysis was conducted for this study. In the analyses of 101 individuals in this paper, we have the same level of power to detect an effect size of *d* = 0.57 in differences between two equal-sized subgroups or a product-moment correlation of 0.27.

Inclusion criteria were age 14 to 26 years, diagnosis of RB, and able to speak and read English. Exclusion criteria included severe developmental delay precluding self-report and/or current diagnosis of second malignant neoplasm.

### Data collection procedures

Institutional review board (IRB) approval (Children’s Hospital Los Angeles [CHLA] IRB # 19–00014 and University of California Los Angeles [UCLA] IRB # 19–000305) was obtained prior to study commencement. Both parent/legal guardian consent and participant assent were obtained for participants younger than age 18, and informed consent was obtained from adult participants. A study information sheet and consent form were read aloud to blind participants or emailed to them for voiceover software use if needed. A witness signature was obtained for adult blind participants. Written and/or verbal assent was obtained from minors who were blind; waiver for participant signature was obtained from the IRB and witness signature obtained for verbal assent.

After consent was obtained, participants completed a demographic form, the Patient-Reported Outcome Measurement Information System (PROMIS®)-29 Profile v 2.1 [10, 11], a single PROMIS overall QOL item [8], and the National Institutes of Health (NIH) Toolbox® VRQOL measure [12] utilizing Research Electronic Data Capture (REDCap®) [13, 14]. Participants independently completed all study measures on an iPad while in clinic with no parental assistance or proxy reports. The principal investigator reviewed surveys upon collection and if there was any missing data (infrequently) asked respondents to complete. As a result, there were no missing items in the dataset. Blind participants used voice over software or completed the surveys with the help of the principal investigator. When COVID-19 occurred, procedures were modified to allow for remote participation. The principal investigator recruited participants via telephone or mailed a letter to their address. Eligible participants completed consent documentation electronically or via mail. REDCap survey invitations were emailed to participants for online completion. A medical chart review was conducted to obtain clinical variables, including age at diagnosis, visual acuity, laterality, heredity, and genetic testing. Participants received compensation and parking validation (if the survey was completed on-site).

## HRQOL and overall QOL measures

### PROMIS®-29 Profile v2.1

The generic HRQOL measure, the PROMIS®-29 Profile v 2.1, includes a pain intensity item and four questions for each of the seven domains: physical function, fatigue, sleep disturbance, pain interference, anxiety, depression, and ability to participate in social roles and activities. Item response theory (IRT) pattern-based T scores were computed (mean of 50 and standard deviation (SD) of 10 in the U.S. general population) [15, 16]. Higher scores represent more of the domain being measured. Thus, higher scores in the functioning domains (e.g., physical function) represent better functioning, while higher scores in the symptom domains (e.g., anxiety) represent worse symptoms [17].

Internal consistency reliability (coefficient alpha) estimates for the PROMIS®-29 domains ranged from 0.77 (sleep disturbance) to 0.94 (pain interference) [11]. Estimated reliabilities for the physical health and mental health summary scores (weighted combinations of physical function, pain, social health, fatigue, sleep disturbance, and emotional distress) were 0.98 and 0.97, respectively [11]. Multiple studies have been conducted that provide evidence of the validity of the PROMIS® measures [18–22]. While the PROMIS®-29 has not been used to date in the RB population, support for the validity of the PROMIS® measures was found in the pediatric oncology population [23].

### NIH toolbox® vision-related quality of life survey

The NIH Toolbox® VRQOL is a 53-item measure with six QOL domains related to visual function: color vision, distance vision, near vision, ocular symptoms, psychosocial well-being, and role performance [12]. The Toolbox domain scores are scored using pattern-based IRT scoring and reported as T scores (mean of 50 and SD of 10 relative to the Toolbox validation sample), with higher scores representing better VRQOL. The Toolbox validation sample had a mean age of 54 (range of 18–85 years) and was similar in gender to the 2010 census data. But it was older, less diverse in terms of race/ethnicity and tended to be less educated. Sixty-seven percent wore glasses or contact lenses for distance vision and 61% wore glasses or bifocals for near vision.

Coefficient alphas for the six scales ranged from 0.85 to 0.94 in the prior study [12]. Product-moment correlations between the National Eye Institute Visual Function Questionnaire (NEI–VFQ) scales and the NIH Toolbox® VRQOL support the validity of this newer measure. Worse NIH Toolbox® VRQOL scores were found among those with self-reported visual deficits [12]. The NIH Toolbox® VRQOL is a newer measure and has not been previously used in adolescent or the RB population. Only one previous study has assessed VRQOL in the adult RB population using the NEI–VFQ [5]. The NIH Toolbox® VRQOL measure was chosen due to the advantages of its IRT developmental basis over classical test theory [24] as well as the applicability of the questions to the adolescent RB population.

### Overall quality of life item

Overall QOL was measured using the second question on the PROMIS® Global Health v1.2 scale [25]. This question states *“In general, would you say your quality of life is?”* with five answer choices ranging from poor to excellent. Correlations between the overall QOL item with other PROMIS® global health items were statistically significant (*p* < 0.001) and ranged from *r* = 0.44–0.70 in the first sample and *r* = 0.36–0.82 in sample two [26].

## Statistical analysis

Demographic and clinical characteristics and VRQOL scores by disease laterality were summarized using mean/median, standard deviation, and range for continuous variables and frequencies and percentages for categorical variables. Differences in baseline characteristics between unilateral and bilateral RB survivors were examined using chi-square tests for categorical variables and Mann–Whitney *U* for continuous variables. Internal consistency reliability (Cronbach’s alpha coefficient) [27] was estimated for the NIH Toolbox® VRQOL and PROMIS®-29 domains. IRT pattern-based T scores for the VRQOL were estimated using the Health Measures Automated Scoring Service and PROMIS® HRQOL scales were estimated using the PROMIS® Assessment Center Scoring Service.^SM^ Normality was assessed with Kolmogorov–Smirnov and Shapiro–Wilk tests. Most measurement domains and clinical variables were not normally distributed. Mann–Whitney *U* tests were used to compare differences in VRQOL between unilateral and bilateral RB survivors.

A correlation of 0.10–0.29 was defined as small, 0.30–0.49 as medium, and > = 0.50 as large per Cohen’s recommendations [28, 29]. We hypothesize medium to large correlations between NIH Toolbox® VRQOL and PROMIS® domains: (1) psychosocial compared to anxiety, depression, and the mental health summary score; (2) role performance with ability to participate in social roles and activities, physical function, and physical health summary score; and (3) distance and near vision with physical function, ability to participate in social roles and activities, and physical health summary scores, respectively. Product-moment and Spearman rank order correlation coefficients are reported.

We examined correlations of the PROMIS® overall QOL item with the PROMIS®-29 and NIH Toolbox® VRQOL scale scores. Next, we regressed the overall QOL item on the PROMIS-29 and Toolbox measures to obtain explained variance. We estimated four models with different subsets of variables: (1) PROMIS-29 domains; (2) VRQOL domains; (3) PROMIS-29 and VRQOL domains; and (4) PROMIS-29 summary scores. We employed five-fold leave-one-out cross-validation with the CVPRESS statistic [30] to identify the subset of variables to include in each model. We report ordinary least squares regression model results for the selected independent variables.

For all analyses, statistical significance was set at a *p* < 0.05. Analyses were performed using the Statistical Package for the Social Sciences (SPSS) Version 26.0 for MAC [IBM; Somers, NY].

## Results

Detailed description of participants including flow diagram of recruitment has been previously published [31]. A total of 184 RB survivors were identified from a clinic database, 61 were unable to be contacted and 123 were screened for eligibility. Of the 123 RB survivors, 7 were screened ineligible, 13 declined to participate, and 2 did not complete the study questionnaires for a total of 101 RB survivors enrolled. We do not have information about the 13 individuals who declined to participate and the 2 who did not complete study requirements, but the study included a racially and ethnically diverse sample with a majority of participants identifying as Hispanic/Latino.

## Demographic and clinical characteristics of unilateral and bilateral retinoblastoma survivors

Among 101 RB survivors, 57% had unilateral disease and 43% had bilateral disease (Table 1). The median age of survivors was 17 with a range of 14–26 years, 50% were female, 65% were Latino/Hispanic, and 27% with middle class socioeconomic status. The median age at diagnosis was 15 months (range 1–108), 88% had an enucleation in at least one eye, and 13% had a previous family history of RB. Out of those who received genetic testing (*n* = 86), 40 (46%) had a germline pathogenic variant in the *RB1* gene. The majority had normal vision defined as > 20/40 in the better seeing eye (86%); 8% were visually impaired and 6% were blind defined as 20/400–20/40 and < 20/400, respectively. There were no statistically significant differences between unilateral and bilateral groups regarding gender, race/ethnicity, and enucleation laterality. Bilateral survivors were older (median 18 versus 16 years) at the time of study but younger (median 9 months versus 20.5) at diagnosis. All unilateral survivors had normal visual acuity while as expected, bilateral survivors showed more family history (23%) of RB and the presence of a pathogenic germline *RB1* gene mutation (74%). Given the small sample sizes for these subgroups any significant difference is non-trivial. For example, the difference in age between the unilateral and bilateral subgroups is a medium effect (*d* = 0.67).

**Table 1:** Demographic and clinical characteristics of retinoblastoma survivors

	Overall (*n* = 101)	Unilateral (*n* = 58)	Bilateral (*n* = 43)	*P*-value^*^
Age at study (years)				
Mean (SD)	17.5 (3.0)	16.7 (2.1)	18.7 (3.7)	**.008**^**a**^
Median (range)	17 (14, 26)	16 (14, 22)	18 (14, 26)	
Gender, *N* (%)				
Male	50 (49.5)	26 (44.8)	24 (55.8)	.373^b^
Female	51 (50.5)	32 (55.2)	19 (44.2)	
Race/ethnicity, *N *(%)				
Latino/Hispanic	66 (65.3)	41 (70.7)	25 (58.1)	.200^b^
White	16 (15.8)	6 (10.3)	10 (23.3	
Black/African American	4 (4)	3 (5.2)	1 (2.3)	
Asian/Pacific Islander	10 (9.9)	4 (6.9)	6 (14)	
Other	5 (5)	4 (6.9)	1 (2.3)	
SES, *N* (%)				
Lower status	14 (14)	6 (10.3)	8 (18.6)	**.019**^**b**^
Lower-middle status	22 (22)	15 (25.9)	7 (16.3)	
Middle status	27 (27)	18 (31)	9 (20.9)	
Upper-middle status	25 (25)	16 (27.6)	9 (20.9)	
Upper status	12 (12)	2 (3.4)	10 (23.3)	
Age at diagnosis (months)				
Mean (SD)	18.7 (17.8)	25.2 (20.4)	10.2 (7.8)	** < .001**^**a**^
Median (range)	15 (1, 108)	20.5 (1, 108)	9 (1, 30)	
Enucleation, *N* (%)				
Unilateral	86 (85.1)	54 (93.1)	32 (74.4)	.112^b^
Bilateral	3 (3)	NA	3 (7)	
Visual acuity, *N* (%)				
Normal	87 (86.1)	58 (100)	29 (67.4)	** < .001**^**b**^
Visually impaired	8 (7.9)	0 (0)	8 (18.6)	
Blind	6 (5.9)	0 (0)	6 (14.0)	
Heredity/family history, *N* (%)	13 (12.9)	3 (5.2)	10 (23.3)	**.017**^**b**^
*RB 1* germline mutation (*n* = 86), *N* (%)	40 (46.5)	8 (13.8)	32 (74.4)	** < .001**^**b**^

Bolded items are statistically significant

*RB* retinoblastoma; *SES* socioeconomic status

*p value between unilateral and bilateral

^*a*^Mann–Whitney test

^*b*^Chi-square

## Internal consistency reliability of VRQOL and PROMIS®-29

Coefficient alphas for the NIH Toolbox® scales in this study were as follows: 0.88 color vision, 0.95 distance vision, 0.93 near vision, 0.84 ocular symptoms, 0.85 psychosocial, and 0.83 role performance. Coefficient alpha for the PROMIS®-29 Profile scales in this study ranged from 0.69 (physical function) to 0.96 (pain interference).

## VRQOL in RB survivors

The VRQOL domain scores in unilateral and bilateral RB survivors are listed in Table 2. Unilateral RB survivors had significantly better VRQOL scores for color vision, distance vision, near vision (all *p* < 0.001), and role performance (*p* = 0.009) than bilateral survivors. Differences in ocular symptoms and psychosocial domains were not significant between the groups. Bilateral survivors scored worse than the NIH Toolbox developmental sample of 819 adults (T score mean = 50) on all domains except ocular symptoms while unilateral survivors scored below the sample mean only on psychosocial well-being and role performance.

**Table 2:** Vision-related quality of life in retinoblastoma survivors

Mean (SD) Median (IQR)				
Scale	Overall (*n* = 101)	Unilateral (*n* = 58)	Bilateral (*n* = 43)	*P* value^*^
Color vision	49.8 (7.9)	53.2 (2.6)	45.3 (10.1)	** < .001**
	53.9 (53.9, 53,9)	53.9 (53.9, 53.9)	53.9 (31.15, 53.9)	
Distance vision	50.4 (12.5)	54.7 (9.0)	44.6 (14.2)	** < .001**
	51.28 (43.27, 59.57)	54.31 (48.07, 64.3)	44.69 (31.1, 53.69)	
Near vision	54.1 (12.1)	58.7 (7.6)	47.7 (14.2)	** < .001**
	58.7 (47.92, 63.37)	60.84 (52.05, 66.65)	48.47 (32.96, 60.92)	
Ocular symptoms	51.4 (9.0)	51.3 (9.0)	51.5 (9.1)	.925
	52.48 (46.27, 61.29)	52.79 (46.45, 61.29)	52.05 (45.69, 61.29)	
Psychosocial	47.3 (8.2)	46.9 (8.5)	47.8 (7.9)	.502
	48.12 (41.69, 57.42)	45.78 (40.11, 57.42)	49.98 (42.15, 57.42)	
Role performance	48.3 (8.3)	50.3 (7.1)	45.7 (9.1)	**.009**
	55.43 (41.61, 55.43)	55.43 (46.59, 55.43)	46.59 (37.81, 55.43)	

Bolded items are statistically significant

Mann–Whitney test

*p value between unilateral and bilateral

## Correlations among the VRQOL, PROMIS®-29, and overall quality of life item

Correlations among the VRQOL, PROMIS®-29, and the overall QOL measures are shown in Table 3. All VRQOL scales were significantly correlated with each other except color vision and ocular symptoms. Most scales had medium to large correlations with each other except color vision which had weak correlations with the ocular symptoms and psychosocial scales. The largest correlation (*r* = 0.838) was between near and distance vision scales. There were large correlations of the PROMIS®-29 physical function scale with the VRQOL color vision (*r* = 0.623), distance vision (*r* = 0.532), and role performance (*r* = 0.570) scales. Role performance also had a large correlation with the PROMIS®-29 ability to participate in social roles (*r* = 0.509). Distance vision (*r* = 0.563), near vision (*r* = 0.527), and role performance (*r* = 0.578) were correlated largely with the PROMIS®-29 physical health summary score. There were medium-sized correlations between the VRQOL psychosocial scale and all PROMIS®-29 scales except sleep disturbance (*r’s* = 0.328-0.488). The overall QOL item had a medium correlation with psychosocial (*r* = 0.391) and role performance scales (*r* = 0.359) and a small correlation with distance vision (*r* = 0.197).

**Table 3:** Correlations between NIH Toolbox® VRQOL and PROMIS®-29 and Overall QOL (*n* = 101)

Spearman correlations						
	CV	DV	NV	OS	PS	RP
NIH TOOLBOX® VRQOL						
Color vision (CV)	–					
Distance vision (DV)	0.596**	–				
Near vision (NV)	0.629**	0.838**	–			
Ocular symptoms (OS)	0.084	0.447**	0.514**	–		
Psychosocial (PS)	0.233*	0.506**	0.476**	0.423**	–	
Role performance (RP)	0.523**	0.645**	0.570**	0.415**	0.630**	–
PROMIS®-29						
Physical function	0.623**	0.532**	0.481**	0.176	0.343**	0.570**
Anxiety	−.076	−.192	−.207*	−.378**	−.357**	−.306**
Depression	−.013	−.204*	−.120	−.316**	−.328**	−.234*
Fatigue	−.111	−.322**	−.399**	−.370**	−.356**	−.303**
Sleep disturbance	0.024	−.150	−.151	−.366**	−.212*	−.178
Social roles	0.323**	0.385**	0.332**	0.314**	0.394**	0.509**
Pain interference	−.273**	−.245*	−.341**	−.404**	−.326**	−.396**
PHS	0.495**	0.563**	0.527**	0.434**	0.488**	0.578**
MHS	0.112	0.320**	0.333**	0.472**	0.413**	0.388**
Overall QOL	0.161	0.197*	0.158	0.176	0.391**	0.359**

Pearson correlations						
	CV	DV	NV	OS	PS	RP
NIH Toolbox® VRQOL						
Color vision (CV)	–					
Distance vision (DV)	0.686**	–				
Near vision (NV)	0.753**	0.876**	–			
Ocular symptoms (OS)	0.112	0.433**	0.482**	–		
Psychosocial (PS)	0.200*	0.504**	0.454**	0.495**	–	
Role performance (RP)	0.537**	0.678**	0.653**	0.493**	0.647**	–
PROMIS®-29						
Physical function	0.718**	0.627**	0.636**	0.198*	0.358**	0.642**
Anxiety	−.126	−.203*	−.219*	−391**	−.369**	−.323**
Depression	−.013	−.159	−.070	−.325**	−.373**	−.262**
Fatigue	−.142	−.290**	−.332**	−.376**	−.374**	−.318**
Sleep disturbance	0.035	−.116	−.088	−.368**	−.201*	−.180
Social roles	0.352**	0.411**	0.402**	0.367**	0.415**	0.557**
Pain interference	−.258**	−.254**	−.318**	−.468**	−.375**	−.387**
PHS	0.695**	0.626**	0.636**	0.279**	0.408**	0.672**
MHS	0.191	0.331**	0.336**	0.495**	0.461**	0.440**
Overall QOL	0.146	0.214*	0.172	0.273**	0.407**	0.357**

*NIH*  National Institute of Health; *VRQOL* Vision-related Quality of Life; *PROMIS* Patient-Reported Outcome Measurement Information System®; *QOL*   Quality of Life; *PHS* physical health summary; *MHS *mental health summary

*p < .05, **p ≤ 0.001

## Overall quality of life on PROMIS®-29 V2.1 and NIH toolbox® VRQOL

Thirteen of 15 correlations of the overall quality of life item with the PROMIS-29 and NIH Toolbox VRQOL scales were statistically significant: depression (*r *= −0.595, *p* < 0.0001), mental health summary score (*r* = −0.575, *p* < 0.0001), anxiety (*r* = −0.465, *p* < 0.0001), ability to participate in social roles (*r* = 0.444, *p* < 0.0001), fatigue (*r* = −0.435, *p* < 0.0001), psychosocial (*r* = 0.407, *p* < 0.0001), sleep disturbance (*r* = −0.386, *p* < 0.0001), role performance (*r* = 0.357, *p* = 0.0003), physical health summary (*r* = 0.321, *p* = 0.0011), pain interference (*r* = -0.312, *p* = 0.0015), ocular symptoms (*r* = 0.273, *p* = 0.0057), physical function (*r* = 0.232, *p* = 0.0110), and distance vision (*r* = 0.214, *p* = 0.0057).

Table 4 provides a summary of the multivariate analysis. The PROMIS®-29 scales explained 38% (adjusted R-square) of the variance in overall QOL, with depression (*ß *= −0.495) and ability to participate in social roles and activities (*ß* = 0.216) having significant unique associations with overall QOL. The mental health summary score was significantly associated with overall QOL (*ß* = 0.575) in the second regression model, explaining 32% of the variance. Among the VRQOL domains, only distance vision, psychosocial, and role performance domains significantly correlated with overall QOL. The psychosocial domain (*ß *= 0.407) was the only VRQOL domain significantly uniquely associated with overall QOL, explaining 16% of the variance. The final model explained 39% of the variance with depression (*ß *= −0.375) and the mental health summary score (*ß* = 0.303) having significantly unique associations with overall QOL.

**Table 4:** Ordinary least squares regression models for overall quality of life on PROMIS®-29 V2.1 and NIH toolbox® VRQOL

Measure	Domain	*ß*	R^2^	Adjusted R^2^	F	*p*-Value
1. PROMIS®-29 Domains	Depression Social Roles	−.495	.391	.378	31.42	< 0.001
		.216				
2. VRQOL Domains	Psychosocial	.407	.166	.158	19.7	< 0.001
3. PROMIS®-29 and VRQOL Domains	Depression Role	−.538	.397	.385	32.28	< 0.001
		.216				
4. PROMIS®-29 PHS & MHS	MHS	.575	.331	.324	48.96	< 0.001

*PROMIS®* Patient-Reported Outcome Measurement System; *NIH* National Institute of Health; *VRQOL* Vision-Related Quality of Life; *PHS* physical health summary; *MHS* mental health summary

When controlling for demographic (age and SES) and clinical variables (medical conditions, enucleation, visual acuity), there was no change to the PROMIS®-29 model. In the second model, SES was significantly associated with overall QOL along with the mental health summary score, explaining 36% of the variance. SES was also significant in the third (VRQOL) model along with the psychosocial domain, explaining 22% of the variance. With SES included, the final model explained 42% of the variance with depression, mental health summary score, and the VRQOL psychosocial domain having unique associations with overall QOL.

## Discussion

### VRQOL in the RB population

The results of this study confirm our hypothesis of worse VRQOL for bilateral compared to unilateral RB survivors. Our findings were like the only previous study that has reported VRQOL in an adult cohort of RB survivors; that study showed that the bilateral group had significantly worse scores than the unilateral group on all scales of the National Eye Institute Visual Function Questionnaire (NEI-VFQ-25) [7]. That study had a self-reported blindness rate of 16%, like our findings of 14% of bilateral survivors with visual impairment/blindness which most likely contributed to the lower VRQOL scores. Friedman and colleagues reported significant differences on domains that we did not find to have significance (e.g., ocular pain, social functioning, and mental health). This variation could be due to the use of different measures. However, high correlations between the NEI-VFQ-25 and the NIH Toolbox® VRQOL have been reported for these domains [12]. Another explanation is that an older historical cohort may not reflect current treatment contributing to more ocular pain (e.g., external beam radiation), some having acquired eye disorders (e.g., cataracts, glaucoma), and visual acuity effects on social functioning and mental health [32].

Our bilateral RB survivors scored worse than the validation sample means on all domains of the VRQOL except ocular symptoms, indicating their HRQOL assessment may be more reflective of vision problems. Unilateral RB survivors scored worse than the sample means on the psychosocial and role performance domains indicating their well-being and functional status may be more affected by visual field deficits. Although the unilateral survivors had normal monocular visual acuity, there is loss of depth perception and peripheral fields because of either enucleation or significant central vison loss in the treated eye. Furthermore, unilateral enucleation early in life results in poor motion processing and oculomotor performance resulting from a lack of visual input to the brain, which can lead to decreased motor function and HRQOL [33, 34]. The cosmetic deformity associated with a prosthetic and its psychosocial impact can affect a child’s self-esteem and willingness to participate in certain social activities, negatively influencing their social and role functions [35, 36].

### Generic versus targeted HRQOL measurements

Our analysis provides support for the NIH Toolbox® VRQOL measure in an adolescent population with visual impairment. The domains showed high levels of internal consistency reliability which were consistent with previously reported findings [12]. We found significant medium to large correlations between all scales of the VRQOL except the psychosocial and ocular symptom domains with color vision. The original psychometric assessment of the NIH Toolbox® VRQOL reported higher correlations for these domains [12]. This finding may reflect an older sample with more acquired ocular diseases increasing the applicability of these three domains. Large correlations indicate the domains may be measuring the same underlying concept and justify the development of an overall score for the measure. Near and distance vision were very strongly associated (0.838), indicating that participants were not able to distinguish the difference between these two domains.

Most correlations between the NIH Toolbox® VRQOL and the PROMIS®-29 Profile scales were medium in size. The PROMIS® physical health summary and the physical function scale had large correlations with the VRQOL color, near and distance vision, and role performance scales, while most correlations with the mental health summary were medium, indicating the NIH Toolbox® VRQOL may be more sensitive to physical than mental HRQOL. One study compared the Short Form Health Survey (SF-36) and the NEI-VFQ and found small correlations even among domains that were measuring the same variable [37].

The medium correlations between the NIH Toolbox® VRQOL and the PROMIS® domains were all consistent with our hypotheses, in addition color vision had medium correlations which we had not hypothesized. Per our findings, the high internal consistency reliability and medium to large correlations support the NIH Toolbox® VRQOL as a reliable measure that adds to the assessment of HRQOL in RB survivors.

## Relationships of VRQOL and PROMIS®-29 with Overall Quality of Life

Four of the 6 NIH Toolbox® VRQOL scales correlated significantly with the single overall QOL item. The magnitude of the associations were smaller than those for the PROMIS-29 scales. This is consistent with the fact that QOL measures often reflect mental rather than physical health [26]. The color vision domain was not significantly associated with overall quality of life. It may not be as applicable in this population due to the sparing of the optic nerve in treatment, although this is not always possible [38]. Thus, color vision deficits in this sample may be due to overall visual impairment or blindness. Distance vision was significantly related to overall quality of life, but near vision was not. This could be because the distance vision scale includes questions related to driving and driving is a key factor in adolescents’ and young adults’ independence. Nonetheless, distance vision was only weakly correlated with overall QOL.

The regression models accounted for no more than 39% of the variance of overall QOL with 61% unexplained or not captured in the RB population. Many of the predictive domains were psychosocial, depression, or mental health related. Our findings could reflect data collection during a global pandemic and civil unrest affecting mental health more pronouncedly in the AYA age group. The results also reflect the conceptual similarity and potential overlap of overall QOL with mental health [39]. The VRQOL appears to not add any additional value to the PROMIS®-29 in predicting overall QOL. While the use of a vision-targeted measure such as the NIH Toolbox® VRQOL does add information in the assessment of HRQOL in AYA RB survivors by incorporating vision-related issues that can impact their daily life, it does not replace the value of generic measures, such as the PROMIS®-29 instrument.

## Limitations

This study adds new information about VRQOL in AYA RB survivors, but it has limitations. Our cross-sectional design precludes the ability to make any causal inferences and limits the interpretation of associations. The lack of a control group for comparison with other types of vision-related disorders limits our ability to make unbiased estimates of effect. The generalizability of our findings may be limited due to our ethnically diverse sample from the Los Angeles community, with most other samples being predominantly Caucasian. Furthermore, our sample reflected the treatment era of a decade ago which may be reflective of the previous use of radiation; additionally, with improvements in treatment modalities there are fewer enucleations required now compared to previous decades. Non-response and selection bias cannot be ruled out as only survivors seeking active follow-up and those who responded to letters and phone calls were recruited. In addition, data were self-reported and with some visually impaired participants requiring assistance in completion of the measures, subjects may have chosen socially acceptable answers rather than being truthful. Moreover, the relatively small sample size limited our options for cross-validation of the regression models. Furthermore, since data collection occurred during the COVID-19 pandemic, psychosocial effects on the AYA population may have played a role in the predominance of mental health factors influencing overall QOL. But perceptions of QOL are largely reflective of mental health so it is not surprising that the PROMIS®-29 and NIH Toolbox VRQOL mental health scales were most strongly associated with overall QOL [39].

## Conclusion

Bilateral RB survivors report worse VRQOL than unilateral survivors, but no differences were found in specific ocular symptoms or psychosocial well-being between groups. Medium to large correlations were found between a targeted measure (NIH Toolbox® VRQOL) and a generic (PROMIS®-29 Profile) HRQOL measures. Our findings indicate that the NIH Toolbox® VRQOL adds to the assessment of HRQOL in AYA RB survivors. Although the NIH Toolbox® VRQOL does not significantly add to the prediction of overall QOL beyond the PROMIS®-29 Profile, it provides information about specific vision-related effects on daily life of AYA RB survivors.

## Data Availability

Not applicable.
